# New findings about atherosclerosis in Brazil from the Brazilian Longitudinal Study of Adult Health (ELSA-Brasil): Novas descobertas sobre aterosclerose no Brasil: Estudo Longitudinal de Saúde do Adulto (ELSA-Brasil)

**DOI:** 10.1590/1516-3180.2016.1344090516

**Published:** 2015-04-14

**Authors:** Paulo Andrade Lotufo

**Affiliations:** 1 MD, DrPH. Full Professor, Department of Internal Medicine, Faculdade de Medicina da Universidade de São Paulo (FMUSP), São Paulo, SP, Brazil.

Do not panic if you confuse "atherosclerosis" and "arteriosclerosis". Few people, including teachers and researchers in the medical world, can describe in few words the difference between these pathological conditions, which have distinct impacts on clinical practice and epidemiological studies. The confusion has become very well established. The American Heart Association publishes a journal called "Arteriosclerosis, Thrombosis, and Vascular Biology" in which most of the articles address the experimental, clinical and epidemiological aspects of atherosclerosis. In contrast, the same association publishes "Hypertension", which has a permanent section on "arteriosclerosis". The differentiation between arteriosclerosis and atherosclerosis comes from one of the fathers of modern cardiology, George Pickering.^1^ I prefer to summarize this discussion with two clear definitions:

1. Atherosclerosis is a complex inflammatory process associated with the presence of oxidized low-density lipoprotein (LDL)-cholesterol in the intima and media of the arterial wall.
2. Arteriosclerosis consists of functional depletion of large-artery elasticity.

My assessment is supported by a recent meta-analysis on 76 studies that revealed that only blood pressure and age correlate with the pulse wave velocity (i.e. arteriosclerosis), thereby explaining more than half of the variability of pulse wave velocity.^2^ On the other hand, it is well known that high cholesterol, hypertension, diabetes and smoking are risk factors for atherosclerosis.

Fifty years ago, Professor Mario Montenegro led a seminal study addressing atherosclerosis in Brazil and the Americas using autopsy cases.^3^^,^^4^ However, subsequently, the approach towards atherosclerosis remained not very well developed in Brazil or elsewhere until the emergence of non-invasive imaging tools. For epidemiological and clinical studies such as the Brazilian Longitudinal Study of Adult Health (ELSA-Brasil), atherosclerosis was assessed through measuring the intima-media thickness (IMT) in the common carotid arteries, either by means of ultrasound or from determination of calcium scores in the coronary artery tree through computed tomography. On the other hand, arteriosclerosis was measured by determining the carotid-femoral pulse wave velocity.^5^^,^^6^^,^^7^^,^^8^^,^^9^^,^^10^^,^^11^^,^^12^^,^^13^

ELSA-Brasil was the first observational study to report in our population both the IMT and coronary artery calcium (CAC), thereby enabling comparison of these measurements in clinical practice.^6^^,^^7^ Moreover, ELSA-Brasil was able to verify associations with migraine,^8^ anxiety/depression,^9^ cognition,^10^ insulin resistance,^11^ inflammatory biomarkers^12^ and neck circumference.^13^

One fascinating finding was the association found in ELSA-Brasil between ideal cardiovascular health as proposed by the American Heart Association and the presence of coronary calcium.^14^ The relationship is not exactly direct and linear (i.e. the higher the frequency of risk factors, the greater the quantity of calcium in the coronary tree also would be). ELSA-Brasil enrolled 4,077 participants with no prior history of cardiovascular disease aged 35 to 74 years who underwent CAC measurement. The ideal risk factors (IRFs) were defined using data on smoking, physical activity, diet, blood pressure, glucose/cholesterol levels and body mass index. Zero IRF score was the worst situation and seven was the best. The distribution of the participants according to the IRFs was: 0 to 1 (n = 1,025; 25.1%), 2 (n = 1,200; 29.4%), 3 to 4 (n = 1,551; 38.1%) and 5 to 7 (n = 301; 7.4%). By comparing the presence or absence of CAC, it could be seen that people with IRF = 2 had a 25% lower frequency of calcium in the coronary arteries. Individuals with IRF = 4 had a 40% lower CAC rate and participants with IRF = 5 to 7, a 50% lower CAC rate. The authors concluded that even among individuals with better cardiovascular health (IRF = 5 to 7), it is possible to find CAC greater than zero. This reflects the notion that IRF measurements do not fully account for all factors that result in coronary artery disease.

These findings will need to be confirmed during the ELSA-Brasil follow-up. However, they indicate that subclinical atherosclerosis markers can play a major role in prevention of cardiovascular disease.
